# The *in vitro* effects of gibberellin on human sperm motility

**DOI:** 10.18632/aging.101963

**Published:** 2019-05-22

**Authors:** Chun-Shuang Xu, Yi Zhou, Zhou Jiang, Li-E Wang, Jiao-Jiao Huang, Tian-Yu Zhang, Yong Zhao, Wei Shen, Shu-Hua Zou, Li-Li Zang

**Affiliations:** 1 Center for Reproductive Medicine, Qingdao Women’s and Children’s Hospital, Qingdao University, Qingdao 266034, China; 2 Institute of Reproductive Sciences, College of Life Sciences, Qingdao Agricultural University, Qingdao 266109, China; 3 The International Peace Maternity and Child Health Hospital, School of Medicine, Shanghai Jiao Tong University, Shanghai 200030, China

**Keywords:** gibberellin, sperm motility, oxidative stress, ATPase activity, AMPK

## Abstract

Gibberellin, a plant growth regulator, is widely used to increase the shelf life and quality of fruits and vegetables. In this study, human semen samples were exposed to different concentrations of gibberellin, which reduced spermatozoa motility *in vitro*. Gibberellin exposure also increased levels of reactive oxygen species and the protein levels of apoptosis markers in human sperm. Gibberellin inhibited the activity of Na^+^/K^+^-adenosine triphosphatase (ATPase) and Ca^2+^-ATPase, which maintain the stability of ions inside and outside the membranes of spermatozoa. Moreover, gibberellin exposure suppressed adenosine triphosphate production and reduced the protein levels of adenosine triphosphate synthases, which may have induced the protein expression of adenosine 5′-monophosphate-activated protein kinase (AMPK) and its phosphorylated form. These results suggest that gibberellin reduces human sperm motility *in vitro* by increasing reactive oxygen species levels and reducing ATPase activity, which may upregulate AMPK and consequently reduce the fertilization potential of spermatozoa.

## INTRODUCTION

Plant growth regulators (PGRs) are commonly applied in agriculture to increase the yield and improve the quality of fruits and vegetables. Gibberellin, one of the most common PGRs, can improve the fruit rate, enable the production of parthenocarpic fruits and increase the harvest index [1]. Recently, gibberellin has been widely used in the cultivation of off-season fruit. The global market for gibberellin is in the range of US $500 million [2], and in China, the annual output of gibberellin is estimated to be over 4000 tons. Although gibberellin is usually considered to be non-toxic, especially when applied in the correct dose for the proper amount of time, high doses or inappropriate applications may have adverse effects on humans and animals. A previous study revealed that gibberellin in the drinking water reduced the body weights and food consumption levels of mice [3]. Gibberellin exposure can cause oxidative stress, increase the levels of malondialdehyde and reduce the levels of anti-oxidant enzymes such as catalase (CAT), superoxide dismutase (SOD) and glutathione peroxidase (GPX) [4, 5]. Furthermore, gibberellin has been found to be hepatotoxic, nephrotoxic, genotoxic, neurotoxic and even carcinogenic and teratogenic [6–9].

Long-term exposure to PGRs may substantially impair male reproduction. The herbicide 2,4-dichlorophenoxyacetic acid, a PGR used globally in agriculture, was reported to reduce sperm motility dose-dependently *in vitro*. Specifically, 2,4-dichlorophenoxyacetic acid reduced the ability of human spermatozoa to penetrate a viscous medium and diminished their capacitation abilities and acrosome reaction rates [10]. Another PGR, 4-chlorophenoxy acetic acid, was found to induce apoptosis in rat gonads [11]. Ethephon, still another PGR, induced chromosomal aberrations (structural and numerical) in spermatocytes and stimulated the expression of γH2AX, an early and sensitive biomarker of genotoxic damage [12, 13]. Gibberellin reduced sperm production and increased the number of immature and abnormal sperm in different model animals [14]. Additionally, gibberellin caused subfertility and even infertility by inhibiting spermatogenesis and impairing testicular tissue [15].

Recently, oxidative stress has been recognized as a major cause of spontaneous male infertility [16]. Environmental pollution can promote reactive oxygen species (ROS) production in male germ cells, and the overproduction of seminal ROS is associated with teratozoospermia and oxidative DNA damage in sperm [17, 18]. The accumulation of active oxygen damages the cell membrane and reduces the activity of the Na^+^/K^+^- and Ca^2+^- adenosine triphosphatases (ATPases), which critically influence sperm motility by maintaining ion homeostasis. Lestari et al. suggested that Na^+^/K^+^-ATPase and Ca^2+^-ATPase activity could predict sperm motility disorder [19]. In addition, adenosine 5′-monophosphate (AMP)-activated protein kinase (AMPK), a protein that regulates energy balance and metabolism, is crucial for maintaining sperm cellular energy homeostasis. In our previous study and this study, we found that the AMPK signaling pathway was associated with sperm motility [20].

Although several studies have investigated the effects of gibberellin on sperm motility in model animals, the impacts of gibberellin on human sperm are not yet understood. Therefore, in the current investigation, we evaluated the effects of gibberellin on the motility of human sperm *in vitro* and explored the underlying mechanisms.

## RESULTS

### Gibberellin exposure reduced human sperm motility in vitro

**Figure 1:**
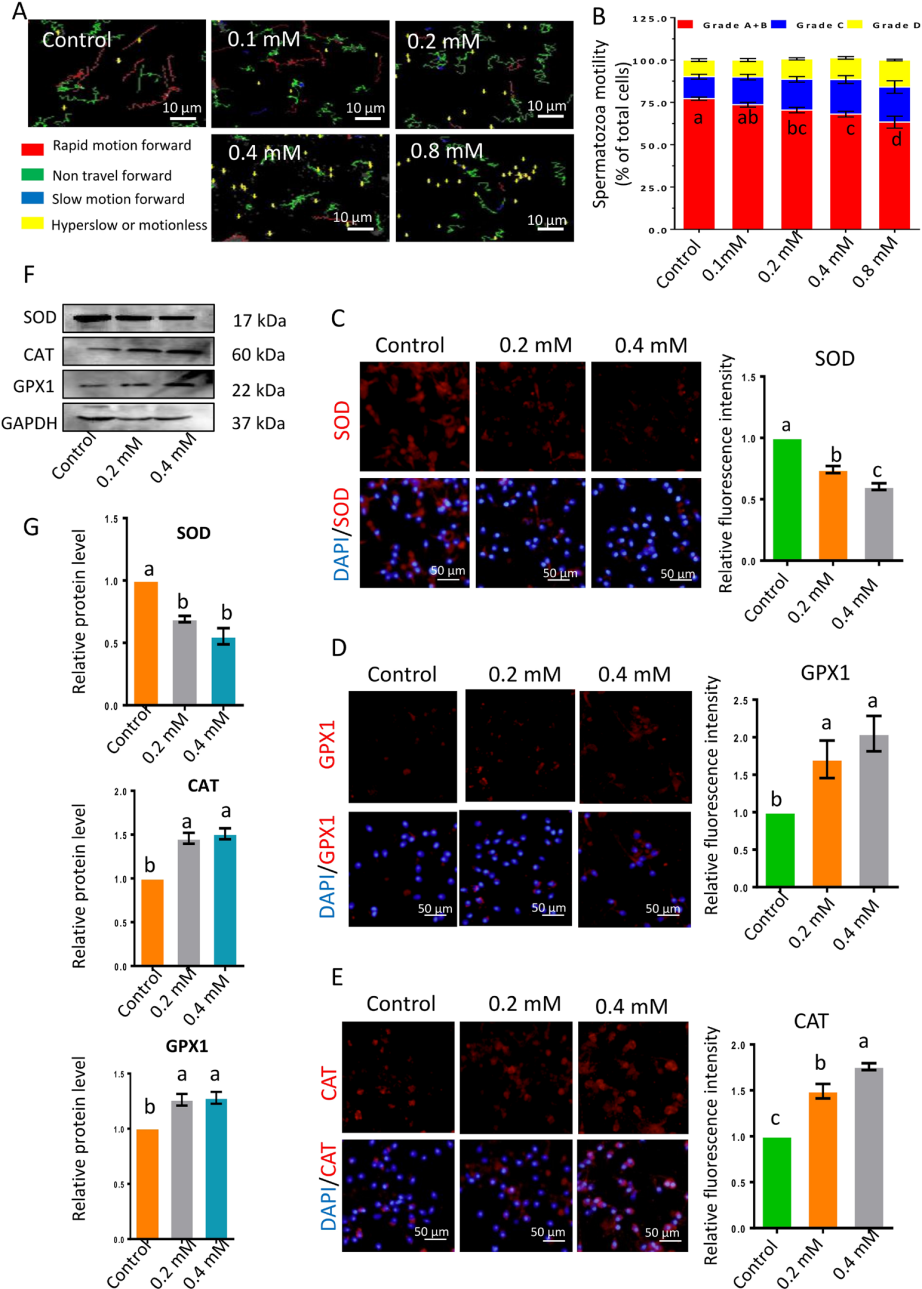
(**A**) Images depicting sperm motility after 1-h exposure to gibberellin. (**B**) Quantitative analysis of sperm motility after gibberellin exposure for 1 h. (**C–E**) The relative protein levels of SOD1, GPX1 and CAT in sperm were detected by immunocytochemistry, and data were quantified based on the relative fluorescence intensity. (**F–G**) The relative protein levels of SOD1, GPX1 and CAT in sperm were detected by Western blotting. The results are presented as the mean ± SEM.

The impacts of gibberellin on sperm were determined at different concentrations and different exposure times (0, 1, 5, 9 and 19 h). Sperm motility was determined with an SCA sperm-class analyzer (Figure 1A and 1B; Supplementary Tables 2 and 3). The motility of sperm exposed to low doses of gibberellin (0.01, 0.04 or 0.08 mM) did not differ from that of the control group; however, high doses of gibberellin (0.1, 0.2, 0.4 and 0.8 mM) dose-dependently reduced sperm motility. Gibberellin exhibited highly acute toxicity at high doses (0.2, 0.4 and 0.8 mM), with a sharp decrease in spermatozoa motility in the first hour *in vitro* (Supplementary Figure 1A; Supplementary Table 3). Therefore, the experimental time was set to 1 h in further analyses.

### Gibberellin exposure increased the ROS levels in human sperm in vitro

**Figure 2:**
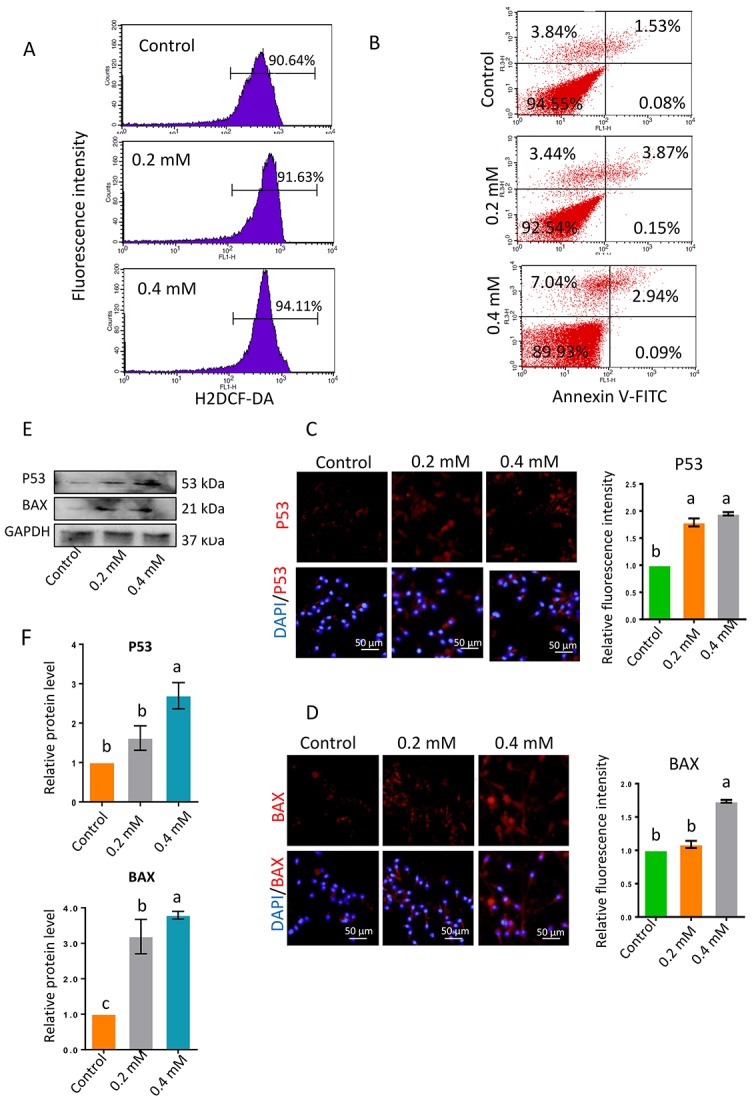
(**A**) ROS levels in sperm determined by flow cytometry with DCFH-DA after 1-h gibberellin treatment. (**B**) Spermatozoon plasma membrane phosphatidylserine externalization, determined by flow cytometry with an Annexin V-FITC apoptosis detection kit. (**C–D**) The relative protein levels of the apoptotic markers BAX and P53 in sperm were detected by immunocytochemistry and quantified based on the relative fluorescence intensity. (**E–F**) The relative protein levels of BAX and P53 in sperm were detected by Western blotting. The results are presented as the mean ± SEM.

As ROS significantly influence sperm motility, the ROS levels in sperm were analyzed by flow cytometry. ROS levels were significantly greater in sperm exposed to 0.2- or 0.4-mM gibberellin for 1 h than in the control group (*P* < 0.05; Figure 2A and Supplementary Figure 1B).

Next, the levels of the anti-oxidant enzymes CAT, SOD and GPX1 were detected by immunocytochemistry and Western blotting. SOD levels were lower in the 0.2- and 0.4-mM gibberellin-exposed groups than in the control group (Figure 1C, 1F and 1G), while CAT and GPX1 levels were significantly increased in the gibberellin-exposed groups compared to the control group (Figure 1D–1G; *P* < 0.05).

### Gibberellin exposure increased apoptosis in human sperm in vitro

To investigate whether apoptosis was involved in the reduction in sperm motility following gibberellin exposure, we used flow cytometry to evaluate the phosphatidylserine externalization of the sperm plasma membrane, a measure of apoptosis in human sperm. Gibberellin exposure induced plasma membrane scrambling, although the level of phosphatidylserine externalization in the sperm plasma membrane remained very low (Annexin V^+^/PI^-^ ratio < 5% of total viable sperm). Exposure to 0.4-mM gibberellin triggered sperm apoptosis (Figure 2B and Supplementary Figure 1C; *P* < 0.01).

In addition, the protein levels of P53 and BAX were detected by immunocytochemistry and Western blotting. The expression of these proteins was significantly greater in sperm exposed to 0.4-mM gibberellin than in the control group (Figure 2C–2F; *P* < 0.01).

### Gibberellin exposure in vitro reduced ATP synthase protein levels and activated the AMPK signaling pathway

**Figure 3:**
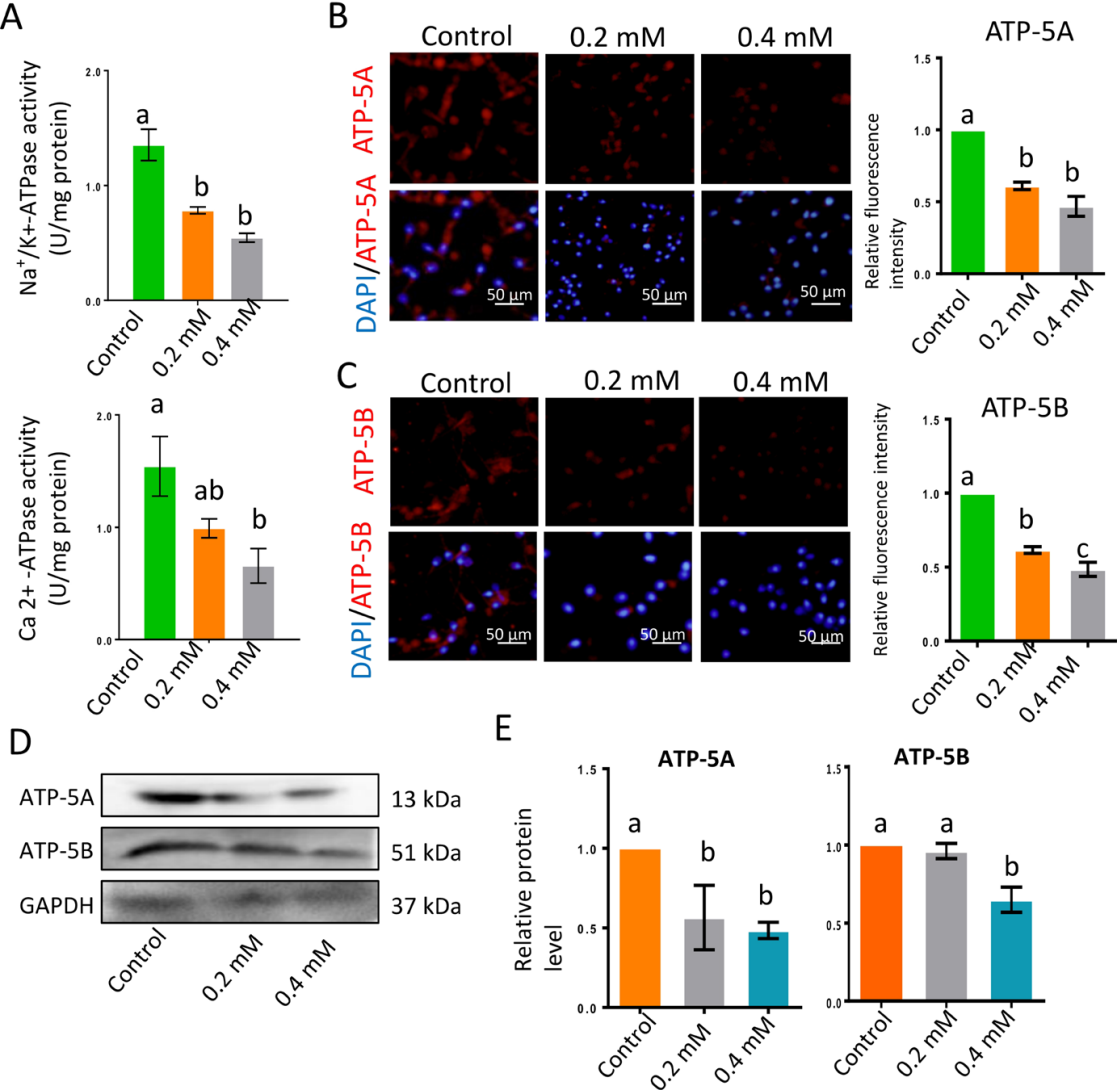
**Gibberellin exposure reduced ATPase activity.** (**A**) Na^+^/K^+^-ATPase and Ca^2+^-ATPase activity in sperm were detected with an ATP assay kit. (**B–C**) The protein levels of ATP-5A and ATP-5B in sperm were detected by immunocytochemistry and quantified based on the relative fluorescence intensity. (**D–E**) The relative protein levels of ATP-5A and ATP-5B in sperm were detected by Western blotting. The results are presented as the mean ± SEM.

Na^+^/K^+^-ATPase and Ca^2+^-ATPase activity are vital for sperm motility. In the current investigation, Na^+^/K^+^-ATPase and Ca^2+^-ATPase activity decreased significantly following gibberellin exposure (Figure 3A and Supplementary Table 4; *P* < 0.01). The protein levels of two major adenosine triphosphate (ATP) synthases, ATP-5A and ATP-5B, were also significantly reduced by gibberellin exposure (Figure 3B–3E; *P* < 0.01). Despite these changes, gibberellin exposure did not alter the population of sperm with a high mitochondrial membrane potential (ΔΨm, Supplementary Figure 1D).

**Figure 4:**
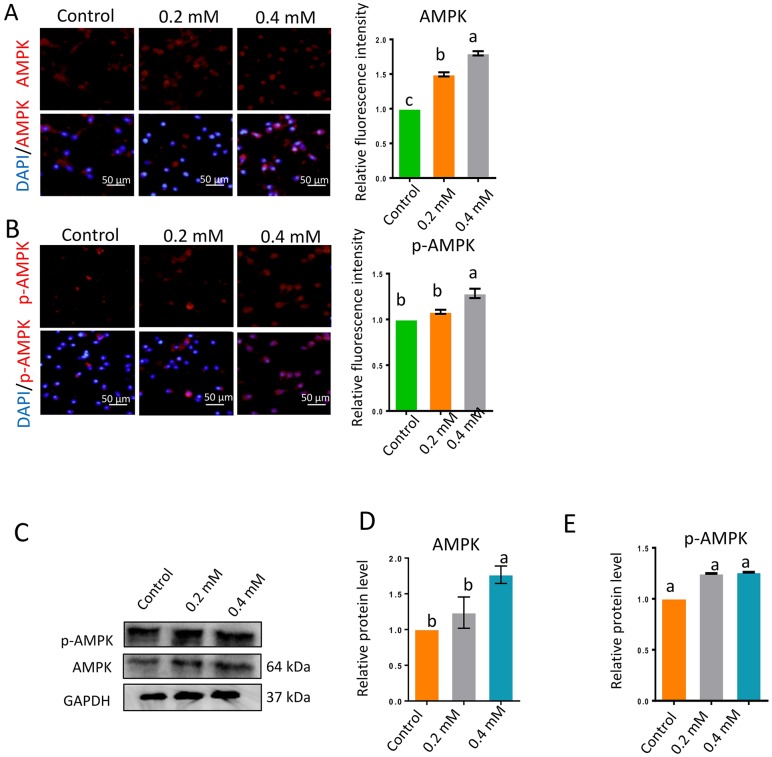
**Gibberellin exposure increased the protein levels of p-AMPK and AMPK.** (**A–B**) The relative protein levels of p-AMPK and AMPK in sperm were detected by immunocytochemistry and quantified based on the relative fluorescence intensity. (**C–E**) The relative protein levels of p-AMPK and AMPK in sperm were detected by Western blotting. The results are presented as the mean ± SEM.

The AMPK signaling pathway is very important for sperm motility and is highly sensitive to the AMP/ATP or ADP/ATP ratio. Inhibited ATP production or increased ATP consumption can reduce the ATP/AMP ratio, thus activating the AMPK pathway by stimulating the phosphorylation of AMPK (p-AMPK). Gibberellin exposure significantly increased the protein levels of AMPK and p-AMPK (Figure 4A–4E; *P* < 0.01).

## DISCUSSION

Recently, many reports have suggested that semen quality is declining all over the world [21–24]. Wang et al. collected sperm samples from 5210 men who were qualified to be sperm donors in China, and found that the sperm volume, sperm motility, semen concentration and total sperm count had all declined between 2008 and 2014 [25]. The reasons for these declines are not fully understood, although studies have indicated that reduced sperm quality might be associated with human papilloma virus and chlamydia trachomatis infections, environmental pollution, drug abuse, smoking, drinking and staying up late [26–31]. The toxicity of gibberellin to the male reproductive system has been a subject of growing concern, and the results of this study confirmed that gibberellin can reduce human sperm motility *in vitro*.

Human spermatozoa are very susceptible to oxidative stress [32], and several studies have demonstrated that the lipid peroxide content of human sperm correlates with severe motility loss [33–36]. Appropriate ROS levels are needed for sperm capacitation, the acrosome reaction and other molecular events in human fertility [37]. However, high ROS levels can break the fluidity and integrity of the sperm membrane, leading to sperm DNA damage and mitochondrial dysfunction [38, 39].

In the current investigation, gibberellin exposure significantly inhibited human sperm motility, increased ROS levels and reduced SOD expression in vitro, indicating that gibberellin induced oxidative stress in human sperm. Although many studies have shown that the increase of ROS will lead to the decreased of CAT and GPXI expression [40–42], some studies have proved that high stress also response to try and increase antioxidant to scavenge ROS [43, 44]. SOD and CAT, anti-oxidant enzymes, help to maintain the stability of the sperm membrane by eliminating oxygen free-radicals. Our study demonstrated that gibberellin disrupted the balance of the oxidation/anti-oxidation system. In other studies, gibberellin exposure significantly reduced anti-oxidant enzyme activity in the kidney, ovary, liver, pancreas and brain [45, 46]. Gibberellin was found to accelerate lipid peroxidation up to 65-fold, leading to oxidative damage [47].

The proper concentration of ions in spermatozoa facilitates sperm flagellar movement. Na^+^/K^+^-ATPase and Ca^2+^-ATPase are important for maintaining the membrane gradient and sperm motility [48, 49]. Under normal physiologic conditions, the CatSper, a ligand-gated Ca^2+^ channel, can be activated by lipophilic compounds. This induces Ca^2+^ inflow and helps to maintain normal sperm motility and function [50]. Lestari et al. demonstrated that Na^+^/K^+^-ATPase activity was lower in men with asthenozoospermia than in those with normozoospermia [51]. Furthermore, Na^+^/K^+^-ATPase and Ca^2+^-ATPase activity are thought to predict sperm motility disorder [52]. The accumulation of active oxygen species can damage the structure of the sperm cell membrane and reduce Na^+^/K^+^-ATPase and Ca^2+^-ATPase activity. Likewise, Ca^2+^ overload can damage and reduce the motility of human sperm.

In this investigation, gibberellin exposure *in vitro* increased the levels of ROS and the expression of the apoptosis-related proteins P53 and BAX. This indicated that gibberellin induced apoptosis in sperm cells. Sperm cell apoptosis is known to be associated with mitochondrial dysfunction [53]. In sperm, mitochondria are likely the major sources of ROS [54]. Oxidative stress promotes the opening of the mitochondrial permeability transition pore, an inner mitochondrial membrane channel, which results in matrix swelling and outer membrane rupture. Consequently, mitochondrial cytochrome C is released, which causes ATP depletion and apoptosis [55, 56].

AMPK is an essential regulatory kinase determining human sperm motility [57, 58], and its signaling pathway is crucial for the maintenance of human sperm cellular energy homeostasis. Reduced ATP levels and increased AMP/ATP or ADP/ATP ratios activate the AMPK pathway. In the current study, the protein levels of two major ATP synthases, ATP-5A and ATP-5B, were significantly reduced by gibberellin exposure *in vitro*. Gibberellin may prevent the formation of ATP, thus increasing AMPK protein expression. Further studies are needed to delineate the toxic effects of gibberellin on sperm mitochondria.

In conclusion, our study demonstrated that gibberellin exposure reduced human sperm motility and Na^+^/K^+^-ATPase and Ca^2+^-ATPase activity while increasing human sperm apoptosis *in vitro*. The AMPK signaling pathway seems to be an important contributor to the toxicity of gibberellin. Investigations are ongoing to determine the effects of gibberellin on other sperm parameters, such as DNA fragmentation and mitochondrial structure and function. Furthermore, it is essential to study gibberellin exposure *in vivo* to determine the concentration of this molecule in the male and female reproductive tracts.

## MATERIALS AND METHODS

### Collection of human sperm samples

This study was carried out at the Center for Reproductive Medicine, Qingdao Women’s and Children’s Hospital, Qingdao, China. 78 ejaculated human sperm samples were collected in sterile containers by masturbation after three to seven days of sexual abstinence from healthy normozoospermic subjects (average age: 32 years old, range: 27-47 years old) living in Qingdao, China. Semen samples were collected in accordance with the World Health Organization guidelines [59]. After complete liquefaction of the semen samples at 37 °C for 30 min, microscopic analysis was performed to determine the following parameters: total sperm motility ≥ 32%, sperm concentration ≥ 1.5×10^7^ cells/mL, sperm viability ≥ 58%, normal sperm forms ≥ 4%, leukocyte concentration < 1×10^6^/mL and lack of sexually transmitted disease (HIV, syphilis and hepatitis B or genital tract infection diseases). In order to minimize individual variation, semen samples obtained from different men were blended for the following test. Each semen donor provided written informed consent for his inclusion in the study. The consent forms and employed methodologies involving human samples were approved by the Ethics Committee of Qingdao Women’s and Children’s Hospital.

### Sperm sample processing, and motility evaluation of sperm exposed to gibberellin

Sperm samples were transferred from sterile containers to 15-mL centrifuge tubes, and fractions of highly motile sperm were separated via discontinuous Percoll gradient (45–90%) centrifugation. These fractions were resuspended at an approximate concentration of 10×10^6^ sperm/mL in G-IVF PLUS medium (Vitrolife, Göteborg, Sweden), which is normally used for the preparation and handling of gametes for *in vitro* fertilization. The resuspended spermatozoa were divided into 2-mL tubes with 1 mL/tube, and three tubes were allocated to each treatment group. The gibberellin (Pribolab Inc., IAC-040-3, Singapore) stock solutions were made by pre-dissolving 100 mg powders in 20 μL 95% ethanol and then diluting them to 0.1mM, 0.2mM, 0.4mM and 0.8mM by deionized water, respectively.

After gibberellin exposure, sperm motility parameters were assessed by computer-aided sperm analysis in accordance with the World Health Organization guidelines (2010). The computer-aided sperm analysis system was operated by an SCA sperm-class analyzer (Microptic SL, Spain). Sperm motility was classified into four types: grade A, linear velocity > 22 μm/s; grade B, linear velocity < 22 μm/s and curvilinear velocity > 5 μm/s; grade C, curvilinear velocity < 5 μm/s; and grade D, immotile. In the clinic, the (A+B)% was used as an index of sperm motility. Sperm samples were stored at 17 °C in a portable incubator (Fuyilian, FYL-19MC-B4, Beijing, China) before motility detection [60]. Prior to the analysis of the gibberellin-exposed samples, the sperm motility of the control group was tested to ensure that the percentage of sperm in grade A + B + C was greater than or equal to 70%. The data were expressed as the mean ± standard error of the mean (SEM).

### Immunocytochemistry

After gibberellin exposure, the samples were washed three times with phosphate-buffered saline (PBS) and fixed in 4% paraformaldehyde for at least 1 h. The sperm cells were then spread on microscope slides and air-dried at room temperature. After three washes with PBS (5 min each), the slides were dipped in 2% Triton X-100 (Solarbio, T8200, Beijing, China) in PBS for 30 min at room temperature. After three washes with PBS, the slides were blocked with PBS containing 10% bovine serum albumin (BSA, Solarbio, A8020) for 30 min at room temperature. Then, the samples were incubated with primary antibodies (1:100; Supplementary Table 1) diluted in blocking solution for 2 h at 37 °C. After three washes with 1% BSA, the slides were incubated with Cy3-labeled goat anti-rabbit IgG (Beyotime, A0516, Nantong, China) (1:150) for 1 h in the dark at 37 °C. The negative controls were incubated with the secondary antibody without the primary antibody.

After three washes with PBS, the slides were incubated with 1 μg/mL Hoechst33342 (Beyotime, C1022) for 5 min at 37 °C. Then, the slides were covered with an anti-fading mounting medium (Vector, H-1000, Burlingame, USA). After the staining, the fluorescent images were viewed through an Olympus microscope BX51 (Olympus, Japan) or a Leica Laser Scanning Confocal Microscope (Leica TCS SP5 II, Germany).

### Western blotting

Total proteins from sperm samples were isolated in radioimmunoprecipitation assay lysis buffer (Beyotime, P0013C) with a protease inhibitor cocktail (Sangon, Shanghai, China) for 30 min on ice. Then, an ultrasonic technique was used to break up the sperm cells, and the total protein concentration was determined with a bicinchoninic acid kit (Thermo Fisher, 23209, USA). Each protein extract was then mixed with sodium dodecyl sulfate-polyacrylamide at a ratio of 1:4 and boiled for 5 min. Then, the lysates were collected by centrifugation (14,000 rpm for 5 min). The total proteins were separated by sodium dodecyl sulfate-polyacrylamide gel electrophoresis with a 3% stacking gel and a 10% separating gel for 40 min at 100 V, followed by 1.5 h at 120 V.

The proteins were transferred to polyvinylidene fluoride membranes at 300 mA for 2 h. Then, the membranes were blocked with 5% non-fat milk (Solarbio, D8340) for 4 h at room temperature, and washed three times with Tris-buffered saline containing 0.1% Tween-20 (TBST). The membranes were incubated with primary antibodies (1:1000 dilution) in TBST containing 1% BSA overnight at 4 °C. The next morning, after three times washes with TBST, the membranes were incubated for 1 h at room temperature with horseradish peroxidase-conjugated goat anti-mouse IgG (Beyotime, A0216) or goat anti-rabbit IgG (Beyotime, A0208) diluted 1:2000 in TBST. After the membranes were washed three times with TBST, a BeyoECL Plus kit (Beyotime, P0018) was used for signal detection. Glyceraldehyde 3-phosphate dehydrogenase (GAPDH) was used as a loading control, and the results were analyzed with AlphaView SA software.

### Flow cytometry analysis

For flow cytometry, a FACSCalibur^TM^ flow cytometer (BD Bioscience, Mississauga, USA) equipped with a 488-nm laser, a forward-scatter diode detector and a photomultiplier tube SSC detector was used. The data were collected with CellQuest software and further analyzed with ModiFit LT (ModiFit LT for Maclntel). About 20,000 sperm were analyzed in each group.

### Annexin V-FITC apoptosis detection kit

Sperm plasma membrane phosphatidylserine externalization (the translocation of phosphatidylserine from the inner to the outer leaflet of the sperm plasma membrane) was detected with an Annexin-V-FITC apoptosis detection kit (TransGen, FA101, Beijing, China) as previously described [22]. After gibberellin exposure, sperm were collected, washed with cold PBS, centrifuged at 1,500 rpm for 3 min and resuspended in 1× Annexin V binding buffer (100 μL). Then, Annexin V (5 μL) and propidium iodide (PI) (5 μL) were added, and the samples were incubated for 15 min in the dark at room temperature. Lastly, 400 μL of binding buffer was added. Samples were analyzed on a FACSCalibur flow cytometer (BD Bioscience) within 30 min. Apoptotic cells were counted and reported as a percentage of the total cell count. The data were expressed as the mean ± SEM.

### Reactive oxygen species assay

ROS were detected with 2,7-dichlorodihydrofluorescein-diacetate (DCFH-DA, Jiancheng, E004, Nanjing, China). After gibberellin exposure, the sperm were stained with DCFH-DA (10 μM) in the dark at 37 °C for 30 mihen, fluorescence was assessed with a FACSCalibur flow cytometer with a 530-nm LP filter. The data were presented as the mean fluorescence intensity ± SEM.

### Mitochondrial membrane potential assay

The mitochondrial membrane potential (△Ψm) was measured with the specific probe JC-1 (Thermo Fisher, M34152). After 1-h gibberellin treatment, the sperm cells were collected and resuspended in 1 mL of PBS. Then, 10 μL of a 200-μM JC-1 solution was added, and the samples were incubated in the dark at 37 °C for 30 min. The fluorescence was assessed in a FACSCalibur flow cytometer with a 525-nm filter, and the percentage of stained cells was recorded as the population of sperm with a high △Ψm. The data were presented as the average percentage of high-△Ψm sperm ± SEM.

### Measurement of Na^+^/K^+^-ATPase and Ca^2+^-ATPase activity

The activity of the Na^+^/K^+^-ATPase and the Ca^2+^-ATPase were determined with a kit from Nanjing Jiancheng Biochemistry Co. in accordance with the manufacturer’s instructions. After 1 h of gibberellin treatment, sperm were collected and lysed in 0.9% NaCl. Then, the enzyme activity in the lysate was determined by the kit, and the optical density was measured with an ultraviolet spectrophotometer (Thermo Fisher, MA, USA) at a wavelength of 660 nm. The protein concentration was determined with a bicinchoninic acid kit (Thermo Fisher, 23209, USA). The data were expressed as the mean ± SEM.

### Statistical analysis

All the experiments were repeated at least three times, and the data were expressed as the mean ± SEM. The data were statistically analyzed with SPSS statistical software (IBM Co., NY). First, the data were evaluated for a normal distribution, and then analysis of variance (ANOVA) was performed. If the ANOVA test was significant, group-to-group comparisons were made with the Least Significant Difference test. Differences were considered significant if *P <* 0.05.

## Supplementary Material

Supplementary Figure

Supplementary Tables
