# Supplementary material for: The in vitro effects of gibberellin on human sperm motility
Source: Aging (Albany NY). 2019 May 22;11(10):3080–93. doi: 10.18632/aging.101963 (PMC6555458; doi:10.18632/aging.101963)
Supplement: Supplementary Figure [file aging-11-101963-s001.pdf]

SUPPLEMENTARY TABLES

Supplementary Table 1. Primary antibodies.

Antibody name	Catalog no.	Molecular weight (kDa)	Source	Company
SOD	bs-10216R	17	Rabbit	Bioss
CAT	bs-6874R	60	Rabbit	Bioss
GPX1	bs-3882R	22	Rabbit	Bioss
P53	ab1431	53	Rabbit	Abcam
BAX	mAb #2772	21	Rabbit	CST
ATP-5A	bs-2435R	13	Rabbit	Bioss
ATP-5B	bs-8600R	51	Rabbit	Bioss
AMPK	bs-1115R	64	Rabbit	Bioss
p-AMPK(Thr172)	bs-4002R	64	Rabbit	Bioss
GAPDH	YM3040	37	Mouse	Immunoway

Supplementary Table 2. The sperm motility (grade A+B) following treatment with low concentrations of gibberellin for different times.

Concentration	Time				
	0h	1h	5h	9h	19h
Control-0.5% ethanol	71.60±1.65	71.99±1.86	69.10±2.31	63.53±1.89	58.19±2.65
0.01 mM	71.03±1.56	72.01±1.80	67.54±1.99	62.26±1.54	57.57±1.94
0.04 mM	71.63±2.51	71.49±2.76	68.12±2.66	63.19±2.42	59.30±2.93
0.08 mM	71.43±1.50	71.59±1.83	67.31±1.89	62.34±2.65	58.68±2.56
P value	0.44	0.72	0.26	0.63	0.71

Supplementary Table 3. The sperm motility (grade A+B) following treatment with high concentrations of gibberellin for different times.

Concentration	Time				
	0h	1h	5h	9h	19h
Control- non-ethanol	76.58±0.95	77.40±2.23	71.59±3.64	67.15±3.29	62.94±1.76
Control-0.5% ethanol	76.88±1.30	77.20±2.64	71.96±4.90	67.38±2.95	63.20±1.71
0.1 mM	76.13±1.23	73.62±3.41	67.39±4.94	61.85±5.72	56.41±5.98
0.2 mM	77.03±1.76	70.49±4.01	65.82±5.06	61.73±5.13	57.45±5.25
0.4 mM	77.30±1.53	68.08±4.08	63.93±4.06	58.78±6.09	53.73±2.70
0.8 mM	75.75±1.37	63.38±6.99	58.84±7.12	55.33±6.05	48.86±3.39
*P*value	0.6	0.00 [a]	0.00 [a]	0.00 [a]	0.00 [a]

[a] Significantly different from the Control-0.5% ethanol ($P < 0.05$)

Supplementary Table 4. Na+/K+-ATPase and Ca^{2+}-ATPase activity (U/mg protein).

ATPase	Concentration			*P* value
	Control-0.5% ethanol	0.2 mM	0.4 mM	
Na+/K+-ATPase	1.35±0.23	0.78±0.05	0.54±0.06	0.001 [a]
Ca 2+-ATPase	1.54±0.46	0.99±0.14	0.65±0.26	0.038 [a]

[a] Significantly different from the Control-0.5% ethanol ($P < 0.05$)
