# Supplementary material for: The in vitro effects of gibberellin on human sperm motility
Source: Aging (Albany NY). 2019 May 22;11(10):3080–93. doi: 10.18632/aging.101963 (PMC6555458; doi:10.18632/aging.101963)
Supplement: Supplementary Tables [file aging-11-101963-s002.pdf]

SUPPLEMENTARY FIGURE

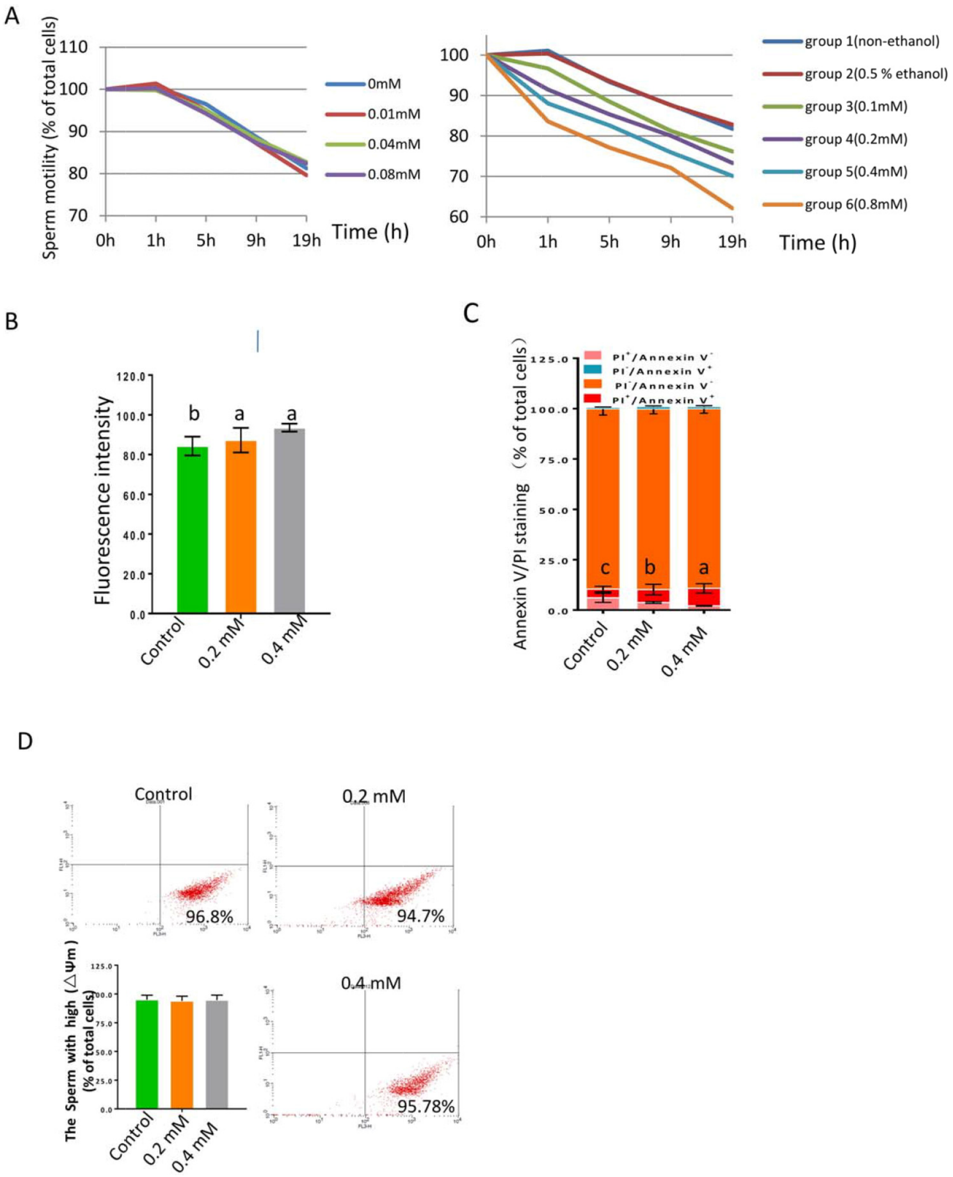

Supplementary Figure 1. (**A**) Quantification of sperm motility after gibberellin exposure for 1, 5, 9 and 19 h. (**B**) Quantitative data on ROS levels after 1-h gibberellin treatment. (**C**) The quantitative data for apoptosis analysis. (**D**) The mitochondrial membrane potential (△Ψm) was measured by flow cytometry with the specific probe JC-1, but the results were not significant. The results are presented as the mean ± SEM.
